# Identification of mangiferin as a potential Glucokinase activator by structure-based virtual ligand screening

**DOI:** 10.1038/srep44681

**Published:** 2017-03-20

**Authors:** Qiuxia Min, Xinpei Cai, Weiguang Sun, Fei gao, Zhimei Li, Qian Zhang, Luo-Sheng Wan, Hua Li, Jiachun Chen

**Affiliations:** 1 Hubei Key Laboratory of Natural Medicinal Chemistry and Resource Evaluation, School of Pharmacy, Tongji Medical College, Huazhong University of Science and Technology, Wuhan, 430030, China; 2 Department of Natural Products Chemistry, School of Traditional Chinese Materia Medica, Key Laboratory of Structure-Based Drug Design & Discovery, Ministry of Education, Shenyang, Pharmaceutical University, Shenyang, 110016, China

## Abstract

The natural product mangiferin (compound 7) has been identified as a potential glucokinase activator by structure-based virtual ligand screening. It was proved by enzyme activation experiment and cell-based assays *in vitro*, with potency in micromolar range. Meanwhile, this compound showed good antihyperglycemic activity in db/db mice without obvious side effects such as excessive hypoglycaemia.

Diabetes mellitus (DM), especially type 2 diabetes mellitus (T2DM), is referred to a metabolic disorder of multiple etiologies in which chronic hyperglycemia results from absent or inadequate pancreatic insulin secretion, with or without concurrent impairment of insulin action^1^. This often leads to carbohydrates, lipids and proteins metabolism disorders, along with serious complications that results in significant disability and mortality^2^.

Glucokinase (GK, EC 2.7.1.2), a glucose phosphorylating enzyme, represents a promising molecular target for development of T2DM drugs. It is an enzyme that facilitates phosphorylation of glucose to glucose-6-phosphate^3^. GK has a pivotal position in the regulation of glucose homeostasis *in vivo*, which acts as a glucose sensor in pancreatic β-cell and a rate controlling enzyme for hepatic glucose metabolism and glycogen synthesis^4^. Studies showed that fifty percent of diabetics had lower liver glucokinase activity than that of the controls^5^. On the other hand, the GK activity can be elevated by glucokinase activators (GKAs), which bind to the allosteric site of GK and contribute to govern blood glucose by enhancing glucose uptake in the liver and potentiating insulin secretion in a glucose-dependent manner^6^,^7^,^8^,^9^,^10^,^11^,^12^,^13^,^14^. Until now, more than 150 patents for GKAs have been recorded^15^,^16^. However, all of them, even the highly promising LY2608204, failed to generate a clinically effective antidiabetic medicine owing to their notable side effect or drug tolerance^17^.

Structure-based virtual ligand screening has been an essential tool in assisting the fast and cost-efficient discovery of lead compounds^18^,^19^. On the other hand, natural products are important sources for drug discovery. They have been an invaluable pool of molecular scaffolds to discover biologically active lead compounds or even new therapeutic agent. Most of currently marketed drugs have been derived directly or indirectly from plant constituent.

In this study, we found that the natural product mangiferin (compound 7, zinc04098535), a C-glycosyl xanthone widely distributed in many plant species, was a potential glucokinase activator by structure-based virtual ligand screening. Previous studies showed that mangiferin could exert effective antidiabetic activities on T2DM animals^20^,^21^,^22^. However, the mechanism of this compound hasn’t been really confirmed. Based on our further study on docking analysis of GK-mangiferin complex, this investigation employed *in vitro* and *in vivo* method to evaluate mangiferin’s antidiabetic potential. All data reported here provide evidence that mangiferin can effectively control postprandial blood glucose levels by moderately activating glucokinase, without the occurrence of adverse effects.

## Results and Discussion

### Structure-based virtual ligand screening

**Figure 1:**
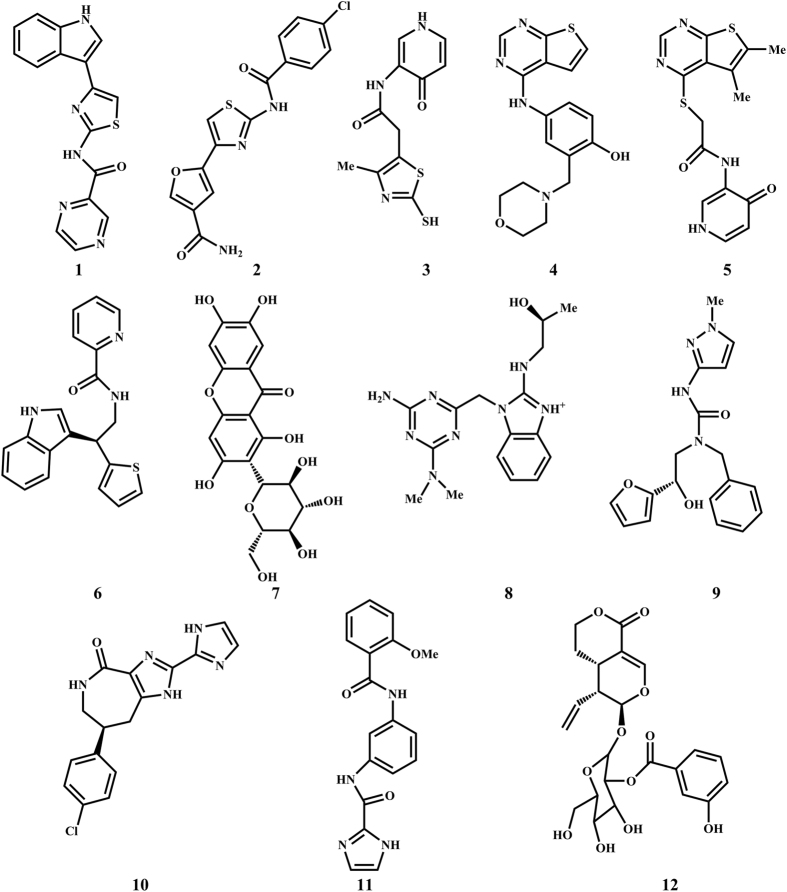
Chemical structures of the top twelve compounds selected by Structure-based virtual ligand screening.

The X-ray co-crystal structure of glucokinase with the allosteric activator (PDB code: 3S41)^14^ was chosen to be the molecular docking model for our study. Based on the model structure of GK, the allosteric site of GK was tri-star shaped. Although tri-forked compounds might bind to the pocket better, this allosteric site can accommodate a variety of chemotype owing to its relatively broad cavities. A total of 6,053,287 lead-like compounds from ZINC^23^ database and 152,056 compounds from the ZINC Natural Products Database were screened according to the internal coordinate mechanics (ICM) method by using ICM-Pro 3.8.1 (Molsoft, San Diego, CA, USA) docking software in silico^18^,^19^. Based on the accessibility of compounds, we selected 12 compounds (Fig. 1) with good ICM scores or mfScores^24^,^25^,^26^, which are also well accommodated into the allosteric site of GK with naked eye observation for further enzymatic evaluation (Table S1).

### The effect of screening hits on GK enzyme activation

**Figure 2:**
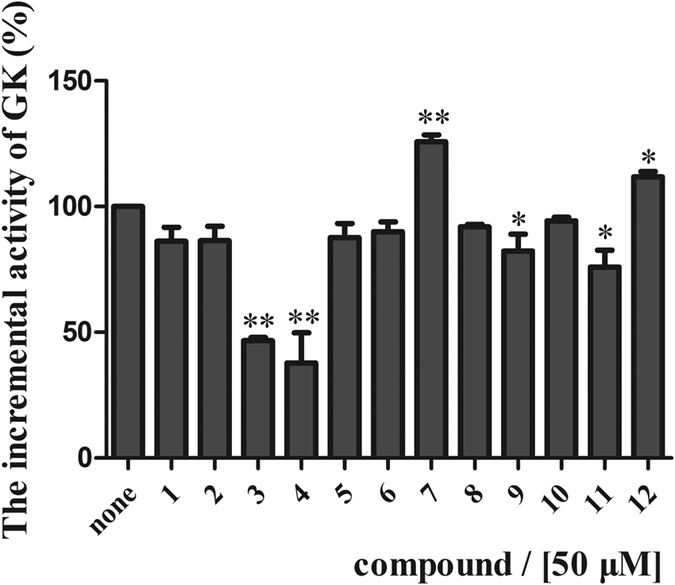
The activation percentage of twelve compounds on GK enzyme activity under the uniform concentration of 50 μM. The activation percentage was the GK enzyme activity in each group compared to that of the blank control group. Each compound was assayed triply. The value presents in a column as the mean ± SD (n = 3). *P < 0.05, **P < 0.01, compared to the blank control group.

In order to further investigate the activation effect of the twelve compounds on GK enzymatic activity, the recombinant GK protein had been successfully obtained by genetic engineering method^4^. Evaluation of the twelve hits on enzymatic activity was assessed spectrometrically by a coupled reaction with glucose-6-phosphate dehydrogenase. Data analysis displayed that both compound **7** and **12** had a positive effect on GK activation (Fig. 2). Furthermore, compound **7** and **12** showed activated effects on GK with EC_50_ values of ca. 156 μM and more than 500 μM, respectively (Table S2).

### Binding affinity assay by microscale thermophoresis (MST)

The binding interaction between GK and compound **7** was assayed by MST, which is a new method that enables the quantitative analysis of molecular interactions in solution at the microliter scale. Results revealed that the binding affinity of compound **7** to GK was 472 ± 20.5 μM, better than that of the known activator of GK, LY2608204 (600 ± 36.1 μM), a potent compound but failed in the phase II clinical trial (Table 1 and Fig. S1).

### Molecular docking analysis of GK-compound 7 complex

**Figure 3:**
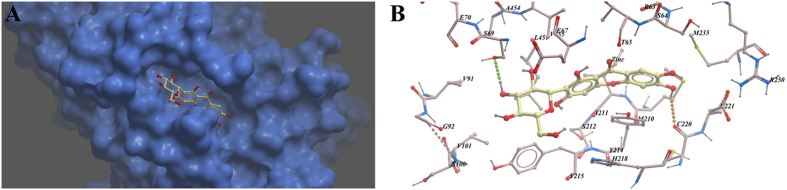
Binding conformations of compound 7 bound to GK generated by virtual ligand docking. Compound **7** depicted as the ball-and-stick model showing carbon (yellow), hydrogen (grey) and oxygen (red) atoms. (**A**) compound **7** was observed to occupy the binding pocket. **(B)** Compound **7** had the ability to form hydrogen bonds with residues Ser69 and Cys220.

To further elucidate the binding mode of compound **7** with GK, we performed molecular docking. The best scoring binding conformation of compound **7** is shown as Fig. 3 In the generated docking model, compound **7** was adopted an extended conformation, which occupied the two major angle of the tri-star shaped allosteric site. Hydrogen bonds were predicted between 4′-OH of sugar ring and Ser69, 6-OH and carbonyl oxygen of Cys220. Moreover, the π-π stacking interaction formed by the pyranone ring of **7** and Tyr214 further strengthens the binding (Fig. 3).

### Glucose consumption in HepG2 and C2C12 myoblast cell

**Figure 4:**
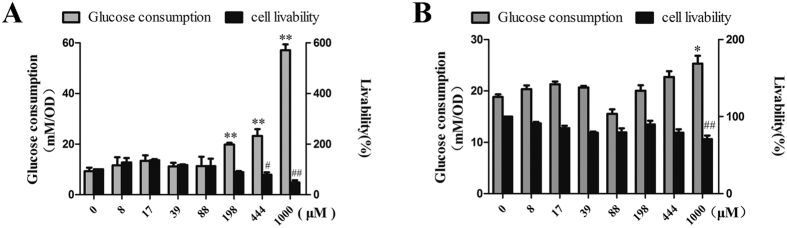
Glucose consumption in HepG2 and C2C12 cell. The glucose consumption was calculated by the reduction of glucose concentrations in culture medium. (**A**) Glucose consumption (grey) and cell livability percent (blank) in HepG2 cell. (**B**) Glucose consumption (grey) and cell livability percent (blank) in C2C12 cell. The cell livability percent in each group was compared to that of the blank control group. Each Sample was assayed triply. Data present in a column as the mean ± SD (n = 3). *P < 0.05, **P < 0.01, glucose consumption level, compared to the blank control group. ^#^*P* < 0.05, ^##^*P* < 0.01, cell livability percent, compared to the blank control group.

HepG2 cell and mouse C2C12 myoblast cell are widely used as the cell models to study hepatocyte and myocyte functions, respectively. In present study, we employed this two cell models to evaluate the effect of compound **7** on cellular glucose consumption. Changes of glucose concentration in culture medium were measured by glucose assay kit, after cells were incubated with various concentrations of compound **7** for 24 hours. As shown in Fig. 4 a remarkable dose-dependent enhancement of glucose consumption of HepG2 cells was observed (Fig. 4A), Meanwhile, compound **7** had a relative slight enhancement on muscle cell glucose consumption (Fig. 4B). This findings suggested that compound **7** could improve glucose metabolism level in HepG2 and muscle cells, especially in the HepG2 cell line. Therefore, compound **7** has a promising potential to govern blood glucose.

### *In vivo* anti-diabetic activities of compound 7 in db/db mice

**Figure 5:**
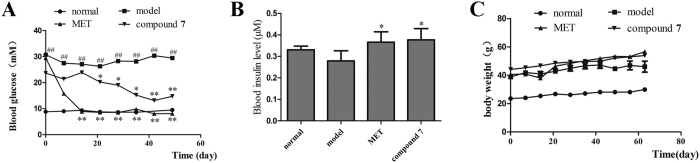
Blood glucose, blood insulin and body weight in mice. (**A**) Blood glucose concentrations measured weekly for 8 weeks. (**B**) Blood insulin level measured at the eighth week. (**C**) Body weight measured weekly for 8 weeks. Data are presented as mean ± SD (n = 6). ^##^*P* < 0.01, compared to the normal group; *P < 0.05, **P < 0.01, compared to the diabetic group. Metformin (200 mg/kg) was used as positive control.

**Figure 6:**
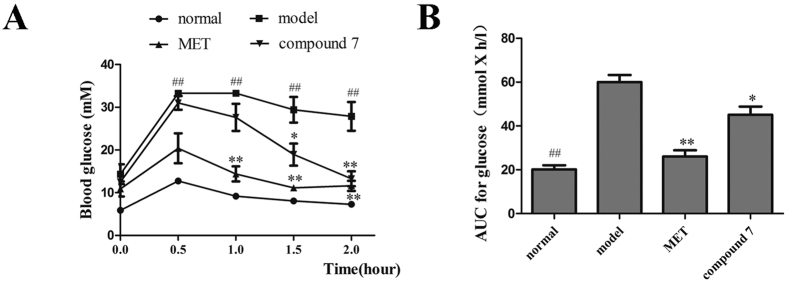
Oral glucose tolerance tests (OGTT) on mice in the eighth week. (**A**) The blood glucose level on oral glucose tolerance tests in mice, (**B**) the AUC of OGTT was calculated. All results above were presented as mean ± SD (n = 6), ^##^*P* < 0.01, compared to normal group; *P < 0.05, **P < 0.01, compared to diabetic model group. Metformin (200 mg/kg) was used as positive control.

After administered with compound **7** (200 mg/kg) for 8 weeks, the two-hour postprandial blood glucose level of **7**-treated group significantly declined, compared with the diabetic group (Fig. 5A). Oral glucose tolerance test (OGTT) was performed at the eighth week. Compared to the diabetic group, both **7**-treated group and metformin-treated group showed palpable hypoglycemia and steady declines (P < 0.05) from 1st to 2nd hour (Fig. 6A). Comparing the area under the curve (AUC) among the groups, metformin and **7** treated groups showed significant reductions, with the degree of 56.55 and 23 41%, respectively (Fig. 6B). The Results revealed that compound **7** improved glucose tolerance in mice. The serum insulin level was determined according to the Mouse Insulin ELISA kit instructions. It showed that the serum insulin level of **7**-treated group had significantly (*P* < 0.05) increases than that of the diabetic group (Fig. 5B). Meanwhile, the serum lipid level in **7**-treated group was slightly increased (Table S3). Results showed that the TG level and HDL-C level of **7**-treated group were obviously lower than that of the diabetic group (*P* < 0.05). The LDL-C level of **7**-treated group was decreasd, but there was no noticeable difference comparing with the diabetic group statistically. The TC level of the **7**-treated group was higher than that of the diabetic group, but there was no significant difference statistically.

### Histopathological examination of tissues

**Figure 7:**
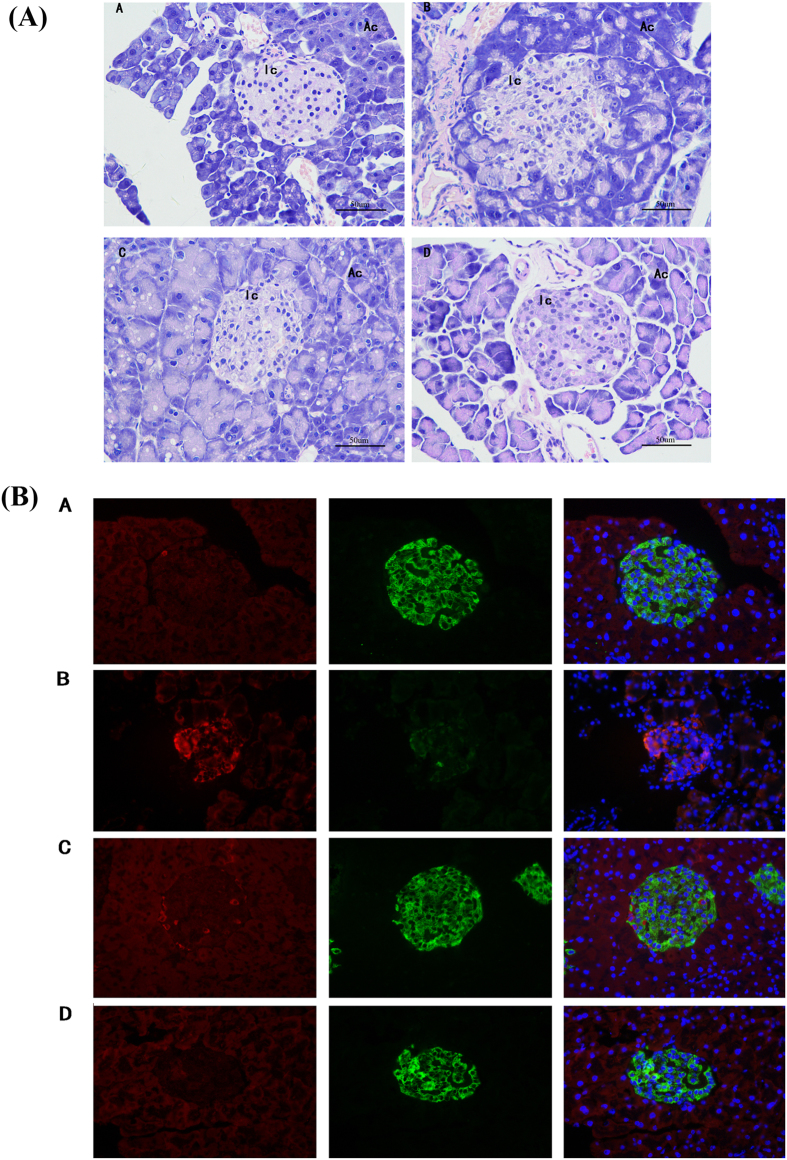
Histological morphology analysis of mice islet tissues. (**A**) Hematoxylin and eosin stained sections of the pancreases of compound-treated groups at the eighth week, Acinar cells (Ac); Islets of Langerhans (Ic); (**B**) Histochemical analysis of insulin and glucagon on islet tissues of compound-treated groups at the eighth week. Pancreatic islets stained for insulin (green flourescence), glucagon (red flourescence) and nucleus (blue flourescence). (A) the normal group; (B) the diabetic group; (C) the metformin-treated group; (D) **7**-treated group. Magnification 400× (DXIT 1200, Nikon, Japan).

The liver lipid deposition has obvious amelioration in compound **7** and metformin treated groups compared to the diabetic group. Histological morphology examination clearly showed adipose accumulation and fat vacuoles in the diabetic group. The abnormal changes were remarkably ameliorated after treatment with metformin and compound **7** (Fig. S2A). Morphology analysis revealed a marked hyperplasia of abdominal white adipose tissue (ABAT) in db/db mice. Compared with the diabetic group, the adipose tissue content and the size of ABAT adipocytes were significantly reduced in the **7**-treated group (Fig. S2B). However, decreased weight gain was unapparent in the **7**-treated group. (Fig. 5C). These findings were consistent with previous results that compound **7** gave rise to the redistribution of adipose depots. Furthermore, the **7**-treated group and metformin-treated group had larger islets with few signs of degeneration, while the diabetic group had smaller islets with grossly disrupted architecture (Fig. 7A). Meanwhile, both two groups had returned to normal expression levels of insulin and glucagen compared to that of the diabetic group (Fig. 7B). This findings suggested that compound **7** might also play a role in the protection and repairation of islet tissues.

In this study, we found the natural product mangiferin (compound **7**, zinc04098535) is a naturally potential agonists of GK by structure-based virtual ligand screening. *In vitro* and *in vivo* experiments have proved that it could effectively control diabetes by moderately activating GK without causing excessive hypoglycemia. All along, people have been searching for the agonist of the GK enzyme. However, none of the drugs has been approved by FDA. The reason is that the activity of GKAs founded currently were too strong to use as a antidiabetic drug. Mangiferin is a common, inexpensive and readily available chemical composition in nature. It could activate the GK enzyme moderately without causing considerable side effect, making this compound as a promising entity for GKAs^27^. And also, more synthetic efforts are required to generate more candidates for *in vitro* and *in vivo* pharmacodynamic evaluation.

## Methods

### Materials

Compound **1, 2, 3, 4, 5, 6, 8**, and **10** were purchased from Enamine Ltd. (Kiev, Ukraine). Compound **7** and **12** were purchased from GuangRun Biotechnology Co., Ltd. (Nanjing, China). Compound **10** and **11** were purchased from ChemBridge Corporation (San Diego, USA). Glucokinase activator LY2608204 (S2155) was purchased from Selleck Chemicals (Houston, TX, USA). Metformin was purchased from Beijing Jingfeng Pharmaceutical Co., Ltd. (Beijing, China). Other chemical reagents were purchased from Sinopharm Chemical Reagent Beijing Co., Ltd (Beijing, China).

### Structure-based virtual ligand screening

The X-ray co-crystal structure of glucokinase with the allosteric activator (PDB code: 3S41)^14^ was chosen to be the molecular docking model for our study. A total of 6,053,287 lead-like compounds from ZINC^23^ database and 152,056 compounds from the ZINC Natural Products Database were screened according to the internal coordinate mechanics (ICM) method by using ICM-Pro 3.8.1 (Molsoft, San Diego, CA, USA) docking software in silico^18^.

### Expression and purification of GK

The full-length human glucokinase (GenBank: BC001890.1) was cloned into the pET-26b vector (Novagen). The recombinant plasmid was transformed into *E. Coli* strain BL21(DE3) (Invitrogen) after verified by sequencing. The recombinant GK proteins were over-expressed and induced by 0.4 mM Isopropyl-β-D-Thiogalactopyranoside (Beijing Dingguo Changsheng Biotechnology Co. Ltd., Beijing, China) at 20 °C for 16 h. Cells were harvested and lysed by ultrasonification on ice in a buffer containing 20 mM Tris (pH 8.0), 200 mM NaCl, and 5 mM β-mercaptoethanol. Soluble C-terminally hexa-histidine tagged GK was purified by a Ni^2+^-chelating column (Qiagen) following by a size exclusion chromatography on a Superdex 300GL column with a buffer containing 20 mM Tris (pH 8.0), 200 mM NaCl. The target protein was finally concentrated to the appropriate concentration by ultra filtration (Millipore).

### Enzymology assay

The *in vitro* efficacy of twelve compounds was assessed in two separate steps: a SC50 assay to evaluate the potency of each compound at a unified compound concentration of 50 μM. Then, we seek to obtain the values of EC_50_ and kinetic parameters Ka, Vmax of promising compounds, according to a reported method with minor modifications^10^. Briefly, Twelve compounds unified in the same concentration of 50 μM were assayed in a 96-well plate with a final reaction volume of 100 μl. The reaction mixture consisted of 25 mM Hepes (pH 7.5), 10 mM glucose, 1 mM ATP, 4 U/ml G6PDH (Sigma-Aldrich Co. LLC., St. Louis, MO, USA), 1 mM NADP, 2.5 mM MgCl, 50 mM KCl, 2 mM DTT, 1 mM recombinant human glucokinase and test compounds. The whole reaction mixtures were kept under 37 °C for 20 minutes. The increase in the rate of absorbance of NADPH generated in the reactions was monitored kinetically at 340 nm. The values were calculated by comparing with untreated GK. This term is called the efficacy of stimulatory concentration (SC_50_). Then, the EC_50_ of promising candidates were determined in the presence of various fixed concentrations of compound **7** (2–300 μM). Values were calculated by fitting enzymatic rates to the Hill equation by using GraphPad Prism 5.01.

### Microscale thermophoresis assay (MST)

The MST assay was performed according to the supplied practice protocol. The recombinant GK protein was labeled with Monolith NTTM Protein Labeling Kit RED (Cat#L001) according to the kit instructions^28^,^29^. Labelled GK concentration was adjusted to 200 nM. A serial of dilution solution of compound **7** (5–10000 μM) in the same buffer (20 mM Hepes, pH 7.5) was prepared and mixed with the above labelled GK protein with the volume ratio of 1:1. After fifteen minutes incubation, all the samples were loaded into the standard glass capillaries. They were immediately measured by MST with a LED power of 100% and a MST power of 40%. The dissociation constrant Kd values were fitted by using NT Analysis software (NanoTemper Technologies, München, Germany).

### Molecular docking

The X-ray co-crystal structure of glucokinase with the allosteric activator (PDB code: 3S41) was used as the docking model. The docking was performed by using ICM 3.8.2 modeling software on an Intel i7 4960 processor (MolSoft LLC, San Diego, CA). Ligand binding pocket residues were selected by using graphical tools in the ICM software to create the boundaries of the docking search. In the docking calculation, potential energy maps of the receptor were calculated using default parameters. Compounds were imported into ICM and an index file was created. Conformational sampling was based on the Monte Carlo procedure^30^, and finally the lowest-energy and the most favorable orientation of the ligand were selected.

### Cell culture

HepG2 cells were purchased from China Center For Type Culture Collection (Wuhan, China). Mouse C2C12 myoblasts were purchased from Shanghai Zhong Qiao Xin Zhou Biotechnology Co. Ltd. (Shanghai, China). HepG2 and C2C12 were cultured in Dulbecco’s modified Eagle medium (High DMEM, GIBCO, USA) supplemented with 10% fetal calf serum (Shanghai Zhong Qiao Xin Zhou Biotechnology Co. Ltd., Shanghai, China), penicillin (100 U/mL), streptomycin (0.1 mg/mL) in a saturated humidified atmosphere of 5% CO_2_ at 37 °C. For differentiation of myotubes, the C2C12 myoblasts were grown until 90% confluency, then changed to differentiation medium for 6 days. The differentiation medium is DMEM containing 2% horse serum (GIBCO, Grand Island, NY), 100 U/ml penicillin, 0.1 mg/mL streptomycin)^31^. The differentiation medium was changed every 24 hours.

### Cell glucose consumption assay

HepG2 and C2C12 myotubes were incubated with compound under various concentrations (0–1 mM) for 24 hours, respectively. The media were collected and glucose surplus was measured at 505 nm with Glu Assay Kit (Shanghai Mind Bioengineering Co. Ltd, Shanghai, China) by Synergy Multi-Mode Microplate Reader (BioTek, USA). Glucose consumption rate was the glucose consumption in each group compared to that of the control group. Cell counting kit-8 (Dojindo, Japan) assay was performed to adjust the value of glucose consumption by calculating the ratio of glucose consumption and CCK-8 (glucose consumption/CCK-8)^32^.

### Animal experimental design

All experiments were performed in accordance with the Guide for the Care and Use of Laboratory Animals of Huazhong University of Science and Technology and approved by the Ethics Committee. The experimental period was eight weeks. Six week-old male db/db mice (B6.BKS(D)-Leprdb/Nju) and lean wild type littermates were purchased from the Model Animal Research Center of Nanjing University. Animals were housed at the animal care facility of Tongji Medical college under standard conditions at constant room temperature of 25 °C, humidity of 60 ± 5%, and on a 12 hours dark/light cycle. Mice had free access to water and food throughout the study period. After 10 days acclimation, diabetic mice were randomly divided into there groups (n = 6). In the experiment, a total of 24 mice (6 normal mice, 18 diabetic mice) were used. Group 1: the normal group, normal mice were orally administered of distilled water (Vehicle); Group 2: the diabetic group, diabetic mice were orally administered of distilled water (Vehicle); Group 3: the metformin-treated group, diabetic mice were orally administered of positive drug metformin (200 mg/kg BW/D) in the same vehicle; Group 4: the **7**-treated group, diabetic mice were orally administered of compound **7** (200 mg/kg BW/D) in the same vehicle.

### Glucose and weight measurements

Weight and two-hour postprandial blood glucose level^33^ of mice were monitored weekly. Glucose measurements were performed on blood drawn from the tail vein using a Bayer Contour Glucose Meter (Bayer, Germany).

### Oral glucose tolerance test (OGTT)

When the db/db mice and normal mice were intragastricly administered for 8 weeks, the 12 h fasted mice in all groups were intra gastricly given glucose (2.5 g/kg). Blood samples were collected from the tail vein at 0, 0.5, 1, 1.5, 2 h after glucose loading, and the blood glucose level of all samples were immediately measured by using a Bayer Contour Glucose Meter.

### Fasting serum insulin levels and lipids levels in mice

The mice were sacrificed after treatment for 8 weeks. Blood samples and tissues for bioassay were obtained from 12 h fasting mice. Blood samples were centrifuged (4000 rpm, 15 min) and then stored at −20 °C for further study. Fasting serum insulin level were measured by the Mouse insulin ELISA kit (TSZ, USA). Total cholesterol (TC), total triglyceride (TG), high-density lipoprotein cholesterol (HDL-C) and low density lipoprotein cholesterol (LDL-C) were respectively analyzed according to the kit instructions, all assay kits were purchased from Jiancheng Bioengineering Institute (Nanjing, China).

### Histopathological examination

The liver, pancreas, abdominal white adipose tissue were removed after the mice were sacrificed. All tissue samples were divided into two halfs, one of which was stored at −80 °C for further study, and the other one were stored in 10% formalin after washing with PBS buffer. The tissues embedded in paraffin and sectioned, and then stained with hematoxylin eosin for histopathological assessment through microscopic observation (Olympus, Tokyo, Japan).

### Statistical analysis

Data are presented as mean ± SD. Statistical analysis was performed using GraphPad Prism 5.0 with one-way analysis of variance (ANOVA). Differences were statistically significant at *P* < 0.05.

## Additional Information

**How to cite this article**: Min, Q. *et al*. Identification of mangiferin as a potential Glucokinase activator by structure-based virtual ligand screening. *Sci. Rep.* **7**, 44681; doi: 10.1038/srep44681 (2017).

**Publisher's note:** Springer Nature remains neutral with regard to jurisdictional claims in published maps and institutional affiliations.

## Supplementary Material

Supplementary Information

## Figures and Tables

**Table 1:** Binding affinity between compounds and GK.

Compound	Dissociation constant with GK
	Kd (μM)
**7**	472 ± 20.5
LY2608204	600 ± 36.1

The dissociation constrant Kd values were automatic calculated by the curve fitting on the NT Analysis software (NanoTemper Technologies, München, Germany). Each compound was assayed triply. The Kd value presents as mean ± SD (n = 3).
