# Supplementary material for: Identification of mangiferin as a potential Glucokinase activator by structure-based virtual ligand screening
Source: Sci Rep. 2017 Mar 20;7:44681. doi: 10.1038/srep44681 (PMC5357792; doi:10.1038/srep44681)

Supplementary information

Identification of mangiferin as a potential Glucokinase activator by structure-based virtual ligand screening

Qiuxia Min[1], Xinpei Cai[1], Weiguang Sun[1], Fei gao[1], Zhimei Li[1], Qian Zhang[1], Luo-Sheng Wan[1], Hua Li[1,2,*], Jiachun Chen[1,*]

a. Hubei Key Laboratory of Natural Medicinal Chemistry and Resource Evaluation, School of Pharmacy, Tongji Medical College, Huazhong University of Science and Technology, Wuhan 430030, China.
b. Department of Natural Products Chemistry, School of Traditional Chinese Materia Medica, Key Laboratory of Structure-Based Drug Design & Discovery, Ministry of Education, Shenyang Pharmaceutical University, Shenyang 110016, China.

Corresponding Author
*Tel: +86-27-83692482. E-mail: homespringchen@126.com.
*Tel: +86-27-83692762. E-mail: li_hua@hust.edu.cn (Hua Li).

Contents of Electronic Supporting Information (ESI)

Tables and figures

Table S1. Docking scores / interaction potential of screening hits

Table S2. The enzymatic efficacy of screening hits on GK

Table S3. Effect of drugs on serum lipids levels in mice

Fig. S1. Measurement of binding affinity between conpounds and GK by MST in standard capillaries.

Fig. S2. Hematoxylin and eosin stained of the (A) liver and (B) abdominal white adipose tissue of groups.

Table S1. Docking scores / interaction potential of screening hits

Compound	ICM docking score (kcal/mol)	ICM docking mfscore (kcal/mol)
**1**	-36.65	-90.83
**2**	-36.06	-95.76
**3**	-30.0876	-37.75
**4**	-33.67	-103.7
**5**	-41.642	-63.2803
**6**	-33.91	-106.5
**7**	-18.3546	-130.8188
**8**	-35.0374	-81.01
**9**	-27.1581	-127.125
**10**	-34.9685	-81.71
**11**	-36.1256	-99.6449
**12**	-22.9813	-135.3

Table S2. The enzymatic efficacy of screening hits on GK

Compound	SC_{50} (%)[a]	EC_{50} (μM)
**none**	100	n.p.
**1**	86.13 ± 5.68	n.a.
**2**	86.46 ± 5.71	n.a.
**3**	46.73 ± 1.21	n.a.
**4**	37.84 ± 12.01	n.a.
**5**	87.63 ± 5.67	n.a.
**6**	90.00 ± 3.88	n.a.
**7**	125.78 ± 2.83	156
**8**	91.95 ± 0.96	n.a.
**9**	82.36 ± 6.72	n.a.
**10**	94.28 ± 1.49	n.a.
**11**	75.92 ± 6.72	n.a.
**12**	111.84 ± 2.14	>500

[a]Data reported as mean ± SD (n = 3). SC_{50} was the activate efficacy of the compound stimulatory concentration of 50 μM. The activity percent of the blank control group (untreated GK group) was setted to 100%.

[b]n.p. is not performed in this study.

[c]n.a. is no activation detected in the experiments.

Table S3. Effect of drugs on serum lipids levels in mice. Each value was expressed as mean ± SD (n = 6); **P* < 0.05, ** *P* < 0.01, compared to the diabetic group. Metformin (200mg/kg) was used as positive control.

Groups	TC (mmol/L)	TG (mmol/L)	LDL (mmol/L)	HDL (mmol/L)
**normal**	14.42 ± 1.31**	4.63 ± 1.85**	0.38 ± 0.14	3.75 ± 0.48
**model**	26.0 ± 2.59	12.64 ± 4.67	0.94 ± 0.48	4.16 ± 0.46
**MET**	27.31 ± 5.64	5.83 ± 1.14	0.39 ± 0.08	3.03 ± 0.20**
**7**	35.39 ± 8.12	0.99 ± 0.20*	0.33 ± 0.07	2.78 ± 0.62*

Fig. S1. Measurement of binding affinity between conpounds and GK by MST in standard capillaries. The binding curve were shown. **(A)** Kd of 472 ± 20.5 μM for compound **7**, **(B)** 600 ± 36.1 μM for compound LY2608204 were calculated.

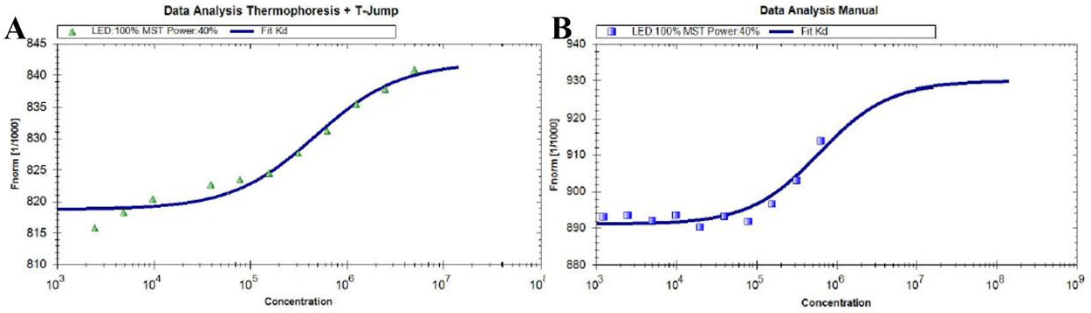

Fig. S2. Hematoxylin and eosin stained of the (A) liver and (B) abdominal white adipose tissue of groups. (A) the normal group; (B) the diabetic group; (C) the metformin-treated group (200 mg/kg); (D) the **7**-treated group. Magnification 200× (liver), 100× (abdominal white adipose tissue) (DXIT 1200, Nikon, Japan).

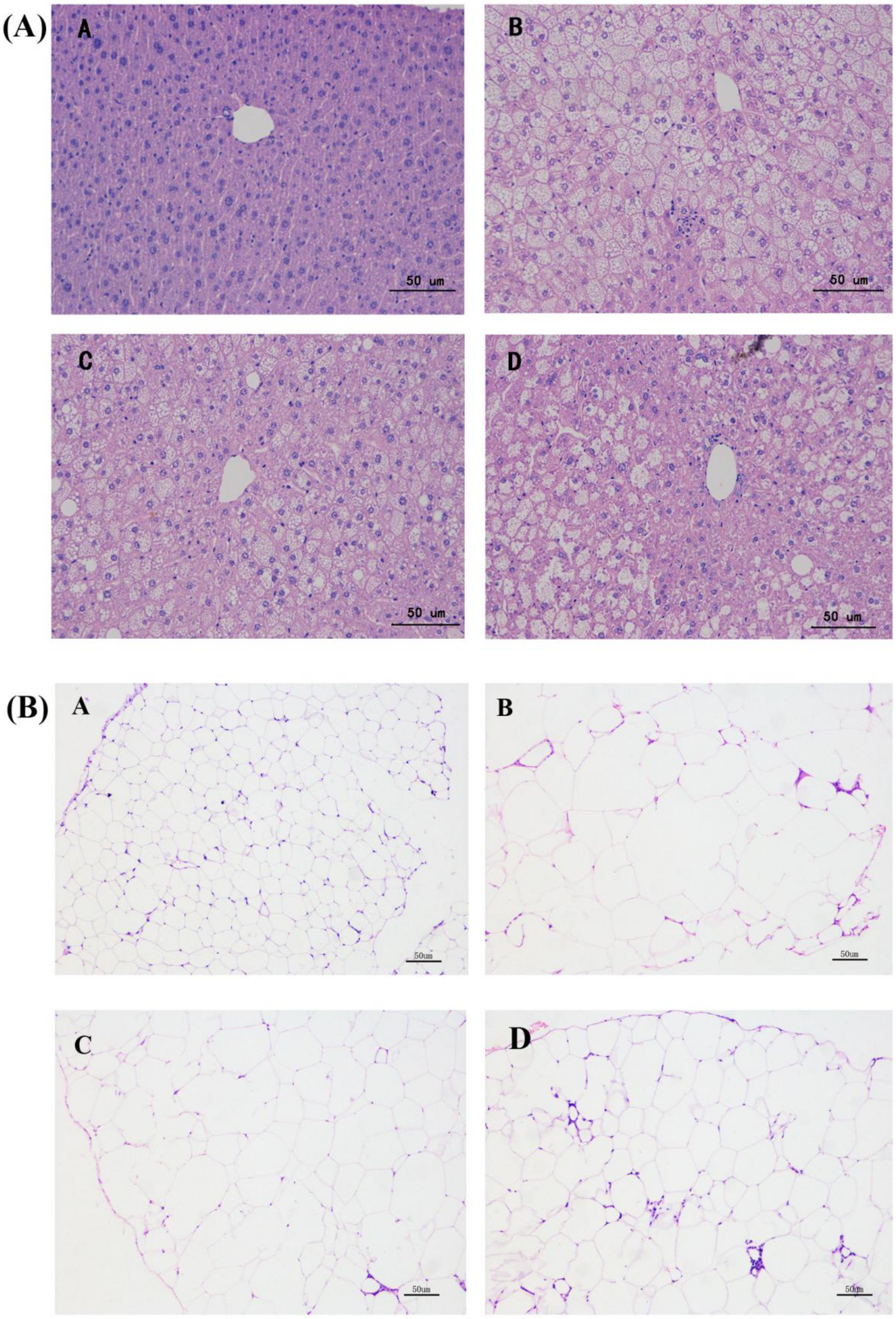

Supplement: Supplementary Information [file srep44681-s1.pdf]
